# Familial Glucocorticoid Receptor Haploinsufficiency by Non-Sense Mediated mRNA Decay, Adrenal Hyperplasia and Apparent Mineralocorticoid Excess

**DOI:** 10.1371/journal.pone.0013563

**Published:** 2010-10-22

**Authors:** Jérôme Bouligand, Brigitte Delemer, Annie-Claude Hecart, Geri Meduri, Say Viengchareun, Larbi Amazit, Séverine Trabado, Bruno Fève, Anne Guiochon-Mantel, Jacques Young, Marc Lombès

**Affiliations:** 1 Assistance Publique-Hôpitaux de Paris, Hôpital de Bicêtre, Service de Génétique Moléculaire, Pharmacogénétique et Hormonologie, Le Kremlin Bicêtre, France; 2 INSERM, U693, Le Kremlin Bicêtre, France; 3 Université Paris-Sud, Faculté de Médecine Paris-Sud, UMR-S693, Le Kremlin Bicêtre, France; 4 Service d'Endocrinologie, Centre Hospitalier Robert Débré, Reims, France; 5 Assistance Publique-Hôpitaux de Paris, Hôpital de Bicêtre, Service d'Endocrinologie et Maladies de la Reproduction, Le Kremlin Bicêtre, France; Leiden University Medical Center, Netherlands

## Abstract

Primary glucocorticoid resistance (OMIM 138040) is a rare hereditary disease that causes a generalized partial insensitivity to glucocorticoid action, due to genetic alterations of the glucocorticoid receptor (GR). Investigation of adrenal incidentalomas led to the discovery of a family (eight affected individuals spanning three generations), prone to cortisol resistance, bilateral adrenal hyperplasia, arterial hypertension and hypokalemia. This phenotype exacerbated over time, cosegregates with the first heterozygous nonsense mutation p.R469[R,X] reported to date for the GR, replacing an arginine (CGA) by a stop (TGA) at amino-acid 469 in the second zinc finger of the DNA-binding domain of the receptor. *In vitro*, this mutation leads to a truncated 50-kDa GR lacking hormone and DNA binding capacity, devoid of hormone-dependent nuclear translocation and transactivation properties. In the proband's fibroblasts, we provided evidence for the lack of expression of the defective allele *in vivo*. The absence of detectable mutated GR mRNA was accompanied by a 50% reduction in wild type GR transcript and protein. This reduced GR expression leads to a significantly below-normal induction of glucocorticoid-induced target genes, FKBP5 in fibroblasts. We demonstrated that the molecular mechanisms of glucocorticoid signaling dysfunction involved GR haploinsufficiency due to the selective degradation of the mutated GR transcript through a nonsense-mediated mRNA Decay that was experimentally validated on emetine-treated propositus' fibroblasts. GR haploinsufficiency leads to hypertension due to illicit occupation of renal mineralocorticoid receptor by elevated cortisol rather than to increased mineralocorticoid production reported in primary glucocorticoid resistance. Indeed, apparent mineralocorticoid excess was demonstrated by a decrease in urinary tetrahydrocortisone-tetrahydrocortisol ratio in affected patients, revealing reduced glucocorticoid degradation by renal activity of the 11β-hydroxysteroid dehydrogenase type 2, a GR regulated gene. We propose thus that GR haploinsufficiency compromises glucocorticoid sensitivity and may represent a novel genetic cause of subclinical hypercortisolism, incidentally revealed bilateral adrenal hyperplasia and mineralocorticoid-independent hypertension.

## Introduction

Glucocorticoids are one of the most important class of steroid hormones that regulate essential biological processes including development, metabolism, growth, inflammatory processes, behavior and apoptosis. Glucocorticoid actions are mediated by the glucocorticoid receptor (GR), a nuclear receptor encoded by the NR3C1 gene [1]. GR is ubiquitously expressed as two distinct isoforms GRα and GRβ. These isoforms are generated by alternative splicing of the last exon 9α or exon 9β of NR3C1 and differ by their last carboxy terminal amino acids and their transcriptional activities [2]. GR regulates a large variety of gene expression in responses to glucocorticoid hormones [3], [4]. A closely related nuclear receptor, the mineralocorticoid receptor also binds glucocorticoid hormones. However, in classical aldosterone target tissues, such as the distal nephron and the colon, the enzyme 11β-hydroxysteroid dehydrogenase type 2 (11βHSD2) metabolizes cortisol into inactive derivative cortisone and prevents permanent MR activation leading to inappropriate sodium retention and subsequent arterial hypertension [5].

To date, twelve germinal mutations of the human GR have been identified; most of them are heterozygous missense mutations causing partial loss of function of the GR [6], [7], [8]. These NR3C1 mutations compromise various steps of the glucocorticoid signalling pathway and cause primary glucocorticoid resistance (OMIM 138040), a rare hereditary disease with generalized partial insensitivity to glucocorticoid action [6]. However, the clinical presentation of primary glucocorticoid resistance is very diverse from severe signs of mineralocorticoid excess due to elevated aldosterone levels, and hyperandrogenia, to mild or asymptomatic forms. This variable phenotype is associated with a wide genetic heterogeneity due to various missense mutations affecting distinct functional domains of the nuclear receptor and responsible for impaired glucocorticoid signalling [9].

Up to date, non-sense mutation of NR3C1 has never been described. We report herein, the discovery of the first heterozygous nonsense mutation in the human GR. We describe a clear-cut clinical, cellular and molecular characterization of a stop mutation and demonstrate that an impaired translation of mutant mRNA *in vivo* causes GR haploinsufficiency. This mutation NR3C1 p.R469[R,X] identified in a French family with eight affected siblings spanning three generations gives a unique opportunity to study the natural history of GR haploinsufficiency as previously reported in mouse models [10]. We thus infer that GR haploinsufficiency in humans is responsible of a discrete phenotype of subclinical hypercortisolism, bilateral adrenal hyperplasia and arterial hypertension. This unusual clinical presentation, most notably incidentally discovered adrenal hyperplasia, progresses gradually throughout the life cycle, and clearly differs from that previously described during primary glucocorticoid resistance associated with other NR3C1 missense mutations. We finally provide evidence that arterial hypertension in this family is related to an illicit MR activation in the kidney, due to altered renal 11βHSD2 activity and hypercortisolism rather than to elevated mineralocorticoids as previously proposed for primary glucocorticoid resistance. Thus, GR haploinsufficiency might be an underestimated cause of bilateral adrenal hyperplasia and arterial hypertension [11].

## Results

### Case Reports

**Figure 1:**
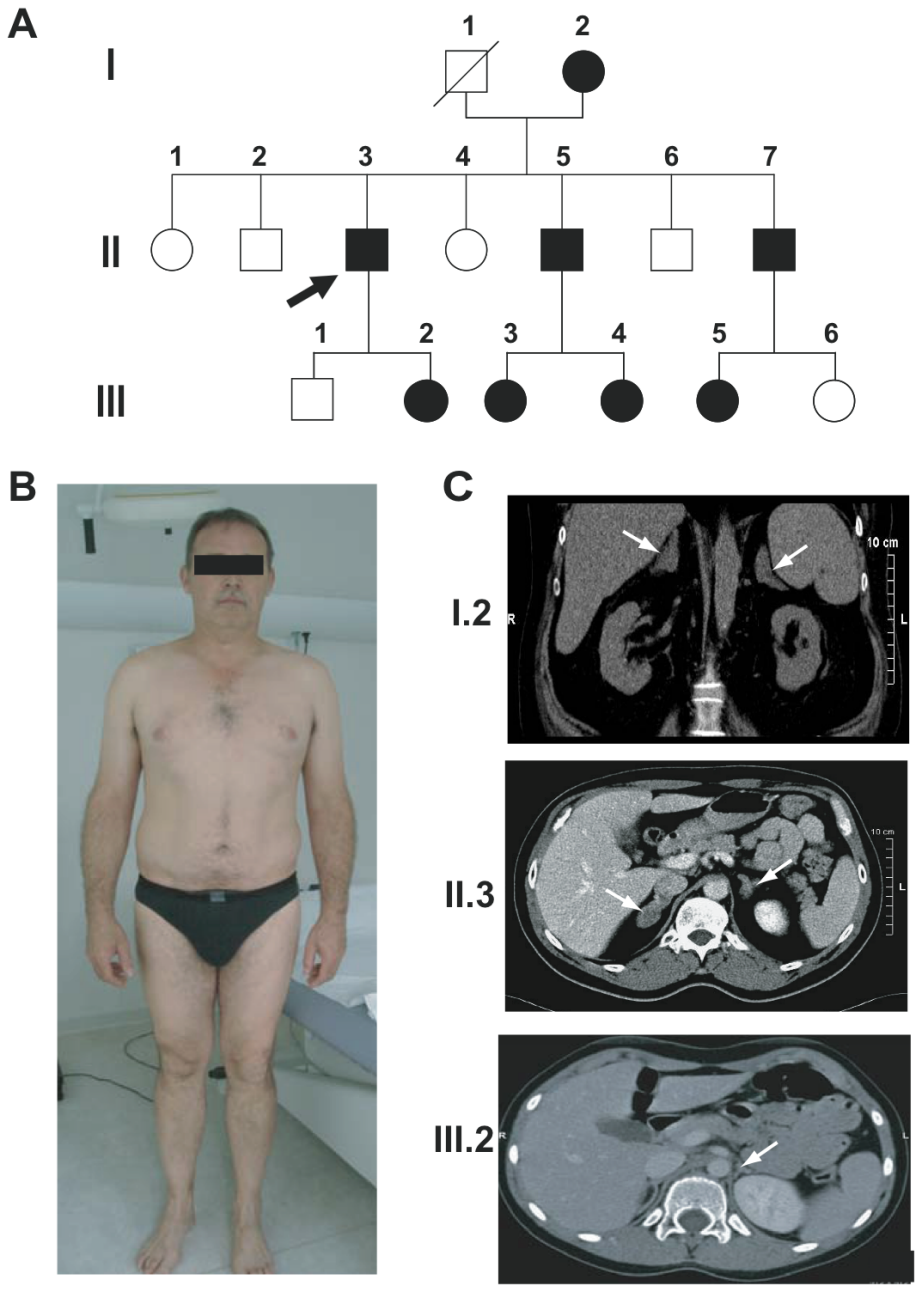
A family with glucocorticoid resistance. **A**) Structure of the pedigree. Three generations (I, II and III) of the kindred are represented. Individuals carrying the heterozygous R469X mutation are shown in black. Black arrow indicates the proband. **B**) Phenotype of the propositus. **C**) Bilateral adrenal hyperplasia was readily visible by computerized tomography (CT) in three affected individuals belonging to three generations (I.2, the mother of the propositus; II.3, the propositus; and III.2, his daughter, white arrows indicate adrenal glands).

The 46-year-old French Caucasian male propositus (subject II.3, Fig. 1A) was referred for evaluation of bilateral adrenal hyperplasia discovered incidentally during computerized tomography (CT) performed for lumbago. His personal history was marked by recent onset of arterial hypertension (190 mm Hg systolic values). Physical examination showed no signs of Cushing's syndrome [12] such as striae bruises, amyotrophy or faciotroncular obesity (Fig. 1B). Biochemical evaluation showed normal glycemia (4.1 mmol/L), moderate hypokalemia (3.5 mmol/L) with inappropriate kaliuresis (55 mmol/d) and normal renal function. Twenty-four-hour urinary free cortisol (UFC) excretion, measured by mass spectrometry-HPLC, was 2- to 4-higher than normal while serum ACTH levels were inappropriate and increased after the CRH test (Table 1). Further endocrine evaluation showed a preserved circardian cortisol cycle but markedly elevated midnight cortisolemia (248 nmol/L). An overnight 1-mg dexamethasone (DEX) suppression test failed to completely suppress plasma cortisol (nadir 119 nmol/L, N <50). Supine aldosterone and active renin levels were undetectable (Table 1) and deoxycorticosterone values were low (40 pg/ml, N 40-200). In contrast, the urinary tetrahydrocortisone/tetrahydrocortisol ratio (THE/THF) was low (0.92, N>1.5), suggesting impaired renal 11β-hydroxysteroid dehydrogenase type 2 (11βHSD2) activity [13]. Abdominal CT revealed bilateral macronodular adrenal hyperplasia (Fig. 1C, middle panel). Normal bone density and osteocalcin levels ruled out detrimental consequences of the cortisol excess on bone structure and metabolism. Magnetic resonance imaging showed normal pituitary and no muscle atrophy and no abdominal or subcutaneous adipose depots.

**Table 1:** Clinical and biological features of the affected and unaffected individuals of the kindred.

#	Sex	Age	BMI (kg/m^2^)	Blood Pressure (Hg mm)	Kalemia	UFC^a^ (µg/d)	F^b^ (nmol/L) DXM sup Test Nadir	ACTH/CRH^d^ (pmol/L)	GR targets Osteocalcin^e^ (ng/mL) THE/THF^f^	Aldo/rennin Aldo^g^ (pg/mL) Renin^h^ (pg/mL) DOC^i^ (pg/mL)
***Affected individuals***										
**I.2**	F	71	34	160/70	3.9 mmol/l*	65 (x2)	Basal 1107Nadir 1236	ND	Osteocalcin 3.5THE/THF 0.72	A<27R 20*
**II.3**	M	46	26	190/100	3.5 mmol/l	94 (x2)	Basal 654Nadir 119	0’ 2.430’ 16.9	Osteocalcin 11THE/THF 0.92	A<27R undetectDOC 40
**II.5**	M	41	30.4	176/102	4.0 mmol/l	192 (x4)	Basal 980	0’ 8.430’ 16.3	Osteocalcin 9.5THE/THF 0.94	A<27R 6.8
**II.7**	M	36	28	120/80	4.0 mmol/l	54 (>N)	Sal F^c^ 48 (x2)Basal 733	0’ 9.215’ 27.1	THE/THF 0.84	A NDR ND
**III.2**	F	9	18.2	96/59	3.6 mmol/l	41 (x2)	Basal 845	0’ 11.7	THE/THF 2.37	A 125R 6.3DOC 76
**III.5**	F	14	20.2	110/60	3.7 mmol/l	124 (x4)	Basal 675Nadir 528	0’ 24.9	THE/THF 2.98	A 37R 12.3DOC 307
***Unaffected individuals***										
**II.1**	F	50	23	115/67	ND	18	Basal 410	ND	THE/THF 1.32	A 71R 2.8
**III.1**	M	16	21	110/70	ND	ND	Basal 413	ND	ND	A 97R 12.8
**III.6**	F	10	17	100/70	ND	33	ND	ND	THE/THF 2.51	A NDR ND

a : Urinary free cortisol, UFC, normal ranges: age>59 y, 7–33 µg/d, 15 to 59 y 13–53 µg/d for males and 11–38 µg/d for females,10–14 y 7–34 µg/d, 5–9 y 4–25 µg/d.

b : Plasma cortisol (F), 8.00 am basal values, normal ranges in adult and children (N<540 nmol/l); overnight 1-mg DEX suppression test, F<50 nmol/L.

c : Salivary cortisol (Sal F), normal ranges 2–19 nmol/L.

d : ACTH, normal values basal 2.2–13.2 pmol/L.

e : Osteocalcin, normal values 5.3–24 ng/mL.

f : THE/THF ratio (Normal values 1.5–2.5).

g : Aldosterone (A), normal resting values 19–117 pg/mL, normal supine values 41–323 pg/ml, 100–800 pg/mL (children).

h : Renin (R), normal resting values, 3–16 pg/ml, normal supine values 3–33 pg/ml, 7.5–25 pg/ml (children)i: deoxycorticosterone (DOC), normal values, 40–200 pg/mL (adult).

* on therapy, ND not determined; NA not available, undetect: below detectable level.

The two affected brothers of the propositus (subjects II.5 and II.7, Fig. 1A) had no clinical features of Cushing's syndrome despite hypercortisolism and insufficiently suppressed plasma or salivary cortisol with low or undetectable renin and aldosterone values (Table 1). The THE/THF ratio was abnormally low in both cases (Table 1). CT also revealed bilateral nodular adrenal hyperplasia in subject II.5 (not shown).

The 72-year-old mother (I.2, Fig. 1A) had a history of severe uncontrolled arterial hypertension and hypokalemia (Table 1), with no clinical features of hypercortisolism or hyperandrogenism (testosterone 0.31 ng/mL, N 0.1-0.4, D4-androstenedione 1.3 ng/mL, N 0.6-1.6, DHEAS 400 ng/ml, N 200-2300). Her bone mineral density was at the upper limit of normal for her age despite probable longstanding glucocorticoid excess. UFC and midnight plasma cortisol levels were elevated and remained high after an overnight DEX suppression test (Table 1). Aldosterone was undetectable. Her THE/THF ratio was also abnormally low (Table 1). CT revealed bilateral nodular adrenal hyperplasia (Fig. 1C, upper panel).

The proband's 9-year-old affected daughter (III.2, Fig. 1A) has normal height, growth and prepubertal development (S2, P1), without clinical hypercortisolism or hyperandrogenism. Her UFC was increased, with inappropriately high ACTH levels (Table 1). Androgen levels were very low. CT also revealed slightly enlarged adrenal glands for her age (Fig. 1C, lower panel). Subject III.5, a 14-year-old daughter of subject II.7, had normal development and regular menses that started at age 12 years. She shows no signs of hypercorticism despite a 4-fold increase in UFC and a negative suppression test (Table 1).

All these clinical and endocrine abnormalities raised the diagnosis of familial generalized glucocorticoid resistance and prompted us to search for genetic defects. We also studied some unaffected family members, including three with normal hormone levels (Table 1) and one with a normal abdominal CT (not shown), in order to demonstrate phenotype and genotype co-segregation.

### Identification of GR mutation and in vitro characterization

**Figure 2:**
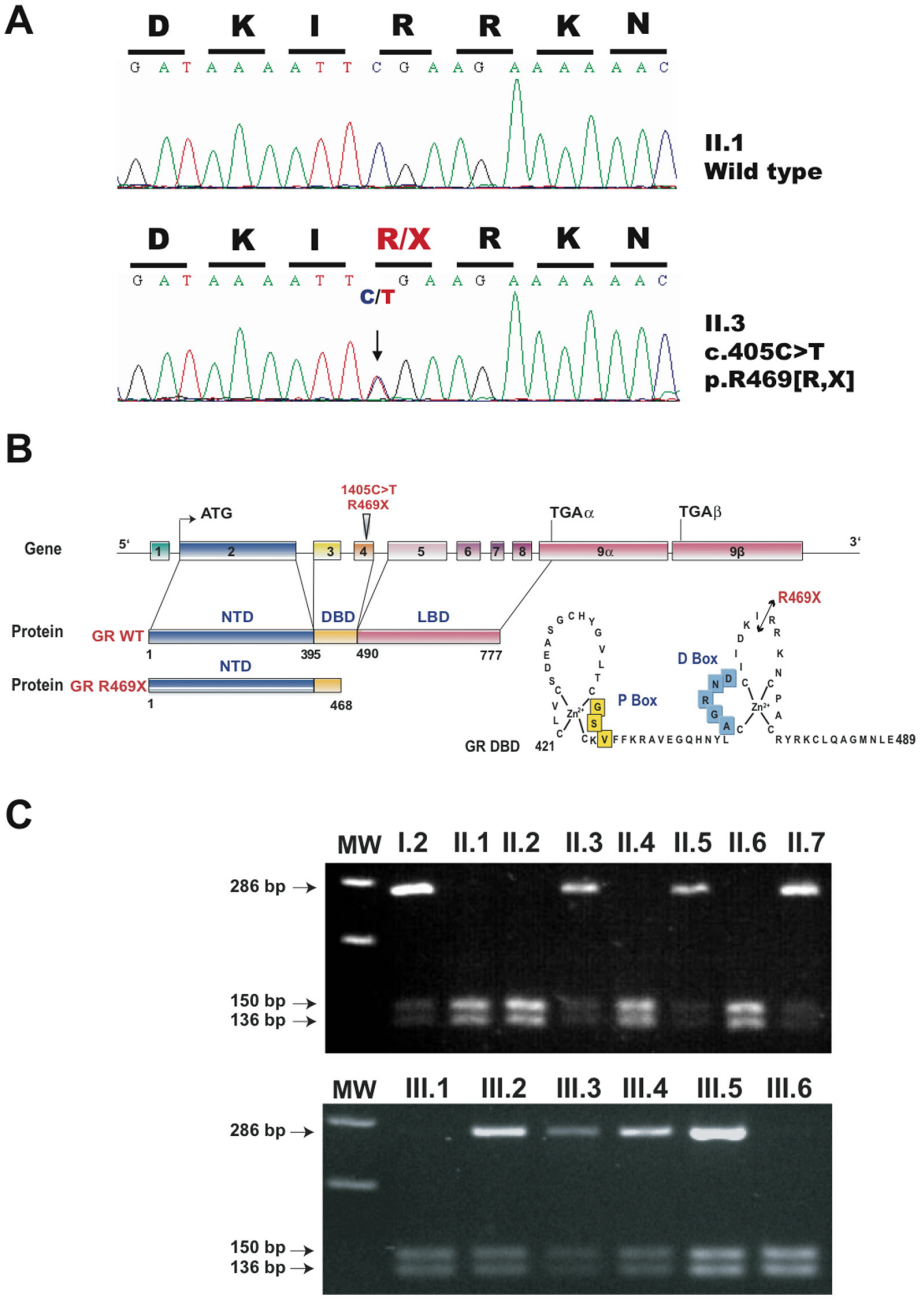
Identification of the GR R469X mutation in the kindred. **A**) Identification of the heterozygous 1405C>T transition. Sequencing of exon 4 in genomic DNA of individual II.1 confirmed the existence of the normal GR coding region, whereas the corresponding sequence of the proband DNA indicated that patient II.3 was heterozygous for a single C>T nucleotide change at position 1405, converting the amino acid arginine (R) into a premature stop codon (X) at position 469 of GR in all affected individuals. **B**) Genomic organization of the human GR and functional domains of the wildtype GRα. The hGR gene is composed of 10 exons, the two last two of which exon 9α and exon 9β are alternatively spliced. The C>T substitution at position 1405 in exon 4 results in a premature termination of translation and gives rise to a 468-amino-acid truncated GR mutant which lacks the C-terminal region of the receptor including part of the DNA-binding domain (DBD) and the ligand-binding domain (LBD). **C**) The 1405C>T substitution abrogated the *Bsp119I* restriction site in exon 4 of GR, thus allowing rapid identification of the heterozygous mutation. PCR-amplified exon 4 fragments from all individuals in the kindred were digested with *Bsp119I* and loaded on agarose gel: (upper panel individuals I.2 to II.7, lower panel individuals III.1 to III.6). The presence of a 286-bp fragment resistant to *Bsp119I* digestion confirmed the C>T substitution.

After sequencing the ten exons and the exon-intron boundaries of the proband's hGR gene, a single heterozygous cytosine to thymidine substitution was identified in exon 4 at nucleotide position 1405 (Fig. 2A), replacing a CGA (arginine) by a TGA stop codon at amino acid 469 in the second zinc finger of the DNA-binding domain of GR. This p.R469X mutation results, as expected, in a truncated GR molecule of 468 amino-acids (Fig. 2B). Interestingly, this nucleotide substitution abrogates the *Bsp119I* restriction site (*TTCGAA*), thus facilitating rapid identification of the mutation in the amplified exon 4 sequence (Fig. 2C). This heterozygous nonsense mutation was detected in eight heterozygous individuals (see Fig. 1A: I.2, II.3, II.5, II.7, III.2, III.3, III.4 and III.5), absent in unaffected family members and in 100 unrelated Caucasians.

**Figure 3:**
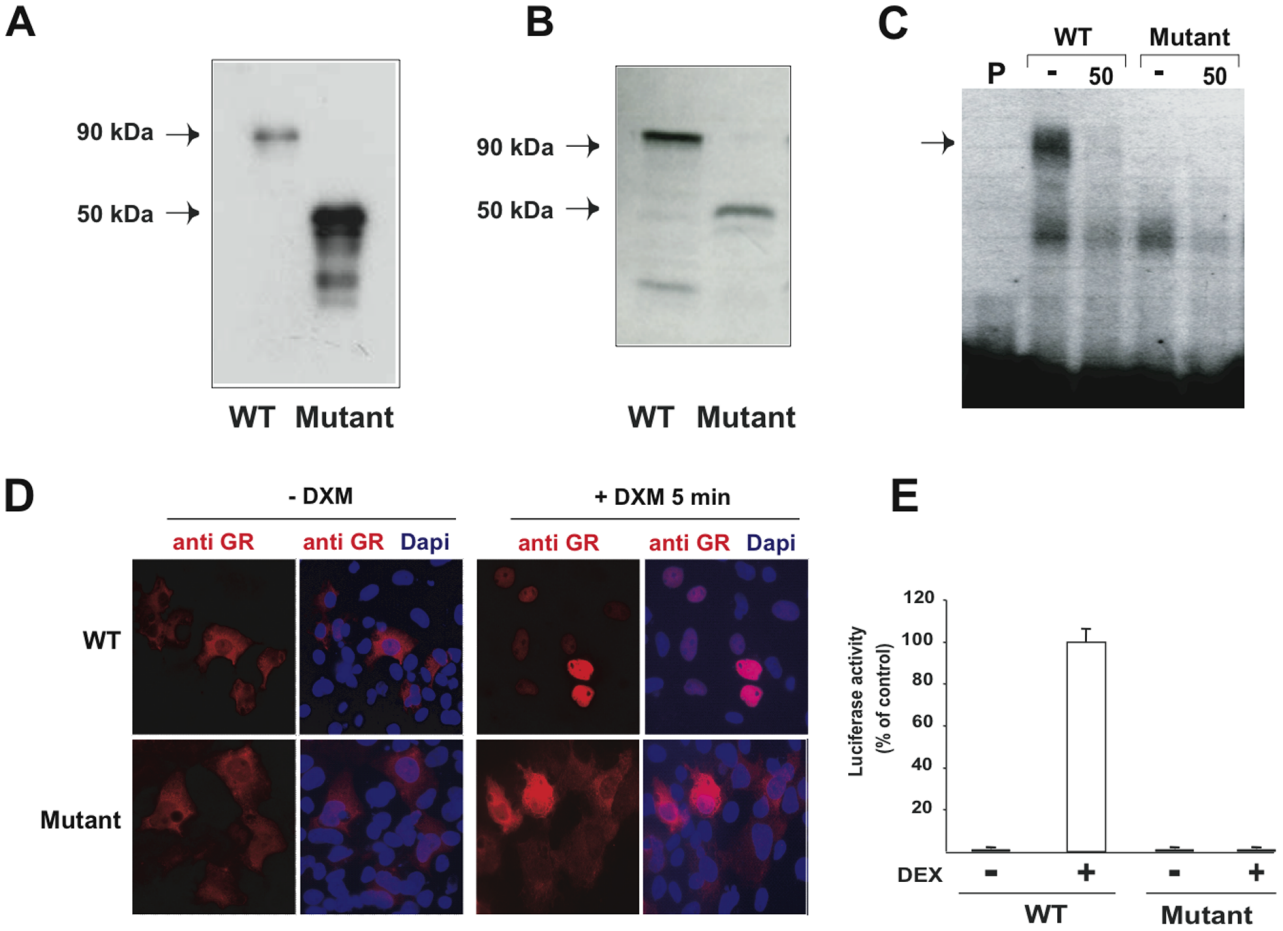
*In vitro* characterization of the GR mutant. **A**) Western blot analysis of GR. Four micrograms of protein obtained from homogenates of HEK293 cells transiently transfected with WT hGRα (WT) and hGRα-R469X mutant were processed for immunoblotting with an anti-GR antibody. Note the presence of a specific 90-kDa band for WT GR and a 50-kDa band for hGRα-R469X. **B**) Protein expression of *in vitro*-translated [^35^S]-labeled WT hGRα and hGRα-R469X separated by 10% SDS-PAGE. GR migrated as a major 90-kDa form whereas the GR mutant, as expected, had a lower molecular mass of approximately 50 kDa. **C**) Binding of *in vitro-*translated WT hGRα and hGRα-R469X to GRE consensus sequence by gel retardation assay. Specific GR-radiolabeled GRE complexes (arrow) were detected in the absence of unlabeled competitor (-), which were abolished in the presence of 50 ng unlabeled probe (50). As expected, the GR mutant was unable to bind DNA. P: free probe. **D**) Intracellular trafficking of WT hGRα and hGRα-R469X in transfected COS7 cells by Immunocytochemistry. Cells were counterstained with DAPI in blue. WT GR translocates from the cytoplasm to the nucleus after 5 min incubation with 1 mM DXM whereas GR mutant remains exclusively in the cytoplasmic compartment either in the absence or presence of DXM. **E**) Transcriptional activity of the WT hGRαand the truncated hGRα-R469X mutant. HEK 293 cells were transfected using Lipofectamin 2000 with either WT hGRα or hGRα-R469X together with the glucocorticoid-responsive reporter gene pGL3-GRE2-TATA-Luc and pSV.β-Gal plasmids. Following transfection, cells were exposed to 100 nM DXM for 24 h. Results (Luc/β-gal activity) are expressed as the percentage of relative transcriptional activity of WT GR arbitrarily set at 100% with 100 nM DXM. Results are means ± SD of at least 3 independent determinations.

The functional properties of the hGRα-R469X mutant were assayed in human HEK 293 cells by transient transfection experiments. As expected from the DBD truncation, the hGRα-R469X mutant migrated as a ∼50-kDa band as shown by Western blot with an antibody recognizing the N-terminal part of GR (Fig. 3A) and by autoradiography of the radiolabeled receptor obtained in a translation-coupled-to-transcription assay (Fig. 3B). The mutant receptor was unable to bind DNA as shown by gel retardation assay (Fig. 3C) neither to translocate to the nucleus after dexamethasone exposure (Fig. 3D). Finally, the mutant was unable to transactivate the GRE2-Luc reporter gene in the absence or presence of hormone (Fig. 3E).

### In vivo characterization of GR mutation and GR Haploinsufficiency

**Figure 4:**
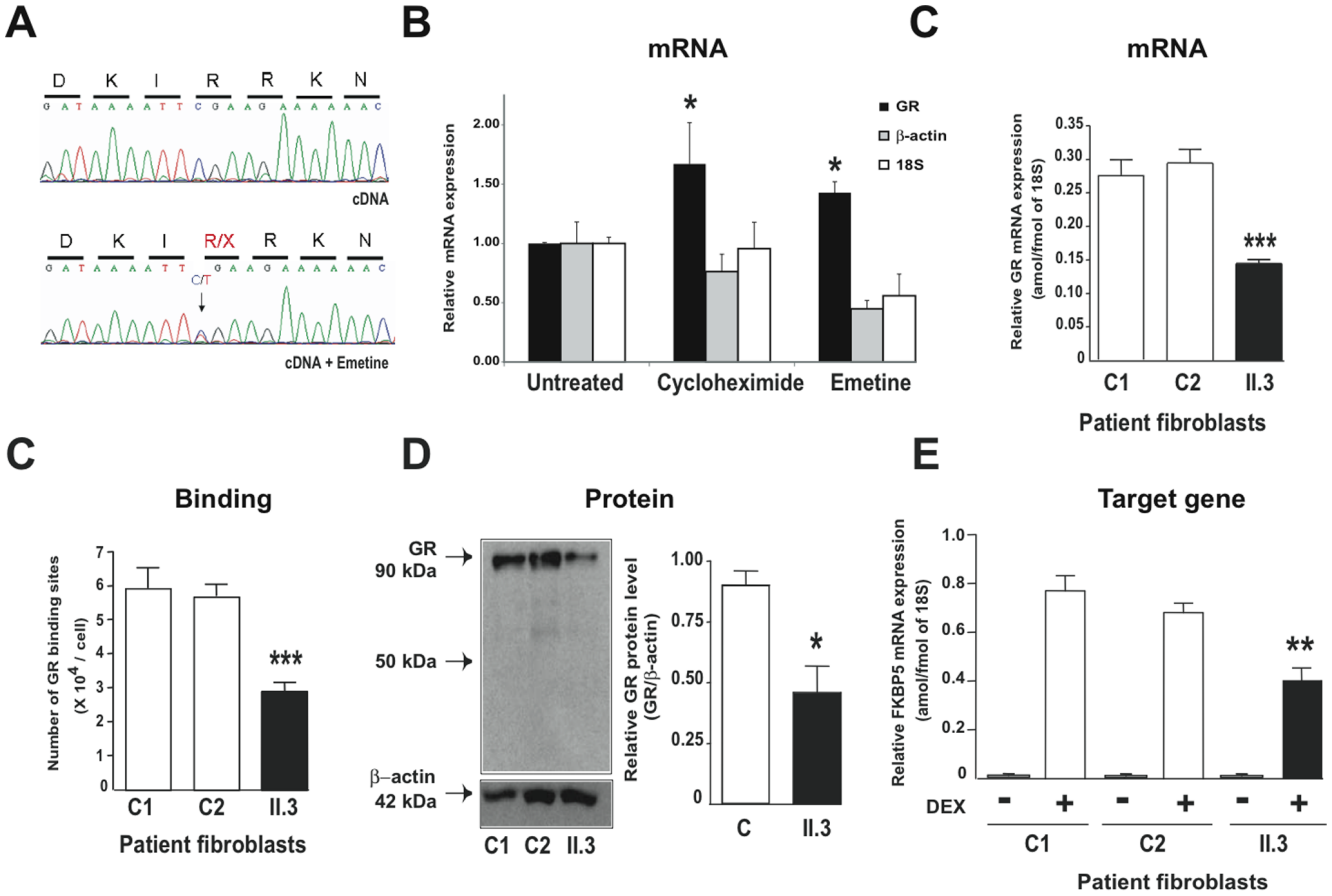
Evidence of GR haploinsufficiency in the proband's fibroblasts due to nonsense-mediated mRNA Decay. **A**) Demonstration of GR haploinsufficiency in the fibroblasts of the propositus (II.3). Sequencing of exon 4 genomic DNA prepared from the patient's fibroblasts confirmed the presence of the heterozygous C>T substitution as observed in lymphocyte genomic DNA (see Supplemental Fig. S1A SI lower panel). In contrast, direct sequencing of the cDNA (see specific primers in Supplemental Table S1 SI) prepared from fibroblast RNA revealed only the wildtype C allele. The absence of mutated GR transcripts (upper panel) in the patient's fibroblasts is consistent with nonsense-mediated mRNA Decay, a cellular mechanism that prevents translation of mutated mRNA bearing a premature termination codon. When fibroblasts were treated for 6 h with 100 µg/ml emetine (lower panel) or for 2 h with 20 µg/ml cycloheximide (not shown), the expression of the defective allele was restored as shown by direct sequencing of the corresponding cDNA fragment. **B**) Increase in GR mRNA expression in fibroblasts of patient II.3 after exposure to two inhibitors of nonsense-mediated mRNA Decay, cycloheximide (20 µg/ml for 2 h) or emetine (100 µg/ml for 6 h). Relative expression of GR, beta-actin ou 18S RNA was measured by using quantitative real-time RT-PCR. Results are means ± SEM of 4 determinations and expressed as fold induction relative to untreated cells. (* P<0.05 Kruskal Wallis followed by Dunn's post test and Mann Whitney test). **C**) Reduction of GR mRNA expression in fibroblasts of patient II.3 compared with two controls C1 and C2. The expression of mRNA was measured by using quantitative real-time RT-PCR. Results are expressed as attomol/fmol of 18S and are means ± SEM of 3 independent determinations (*** P<0.001 Mann Whitney test). **D**) Reduction in specific [^3^H]-DXM binding sites. Fibroblasts pre-incubated in steroid-free medium for 24 h, were exposed to 50 nM [^3^H]-DXM in the absence or presence of a 500-fold excess of unlabeled DEX for 1 h at 37°C. Radioactivity was measured and specific binding was calculated. Data are means ± SEM of 3 independent determinations performed in triplicate. The estimated GR density in the propositus' fibroblasts was 3×10^4^ sites per cell (*** P<0.001 *vs* controls). **E**) Western blot analysis of GR. Thirty micrograms of protein from fibroblast homogenates of controls (C1 and C2) and patient II.3 were processed for immunoblotting with anti-GR (upper panel) and anti-b actin (lower panel). Note the presence of a specific 90-kDa GR in controls and an approximately 50% reduction in WT GR expression in patient II.3 whereas the 50-kDa band corresponding to the truncated hGRa-R469X mutant was not detected. Quantitative analysis of GR signals normalized to b-actin loading was performed using QuantityOne software (Biorad). Results are means ± SD of at least 3 independent analyses (* P<0.05 Mann Whitney test). **F**) Altered glucocorticoid-inducible gene expression in the patient's fibroblasts. Fibroblasts from controls (C1 and C2) and from patient II.3 were starved for 24 h in steroid-free medium and then exposed to 100 nM DXM for 6 h. Relative levels of FKBP5 transcripts were determined by quantitative real-time RT-PCR analysis. Results are expressed as attomol/fmol 18S and are means ± SEM of 6 independent determinations (** *P*<0.01, Kruskal Wallis followed by Dunn's post test).

Fibroblasts cultured from a proband skin biopsy sample were used for molecular characterization of the endogenous GR mutation *in vivo*. The presence of the heterozygous GR mutation in genomic DNA from fibroblasts of individual II.3 was confirmed. However, direct sequencing of cDNA failed to detect any mutated transcripts (Fig. 4A, upper panel). This was consistent with selective degradation of the mutated GR mRNA through a nonsense-mediated mRNA Decay (NMD), a specific quality-control mechanism that eliminates aberrant mRNAs harboring a premature termination codon before the last exon [14]. The involvement of this active process was unambiguously demonstrated as treatment of patient fibroblasts with either emetine or cycloheximide, two potent NMD inhibitors [15], stabilized the mutant mRNA expressed from the defective allele (Fig. 4A) and significantly increased the total amount of GR mRNA levels as measured by quantitative real-time PCR (Fig. 4B). Furthermore, GR mRNA levels expressed in patient fibroblasts were approximately half those measured in two controls (Fig. 4C). DEX binding assays accordingly demonstrated a 50% decrease in the number of fibroblast hormone-binding sites as compared to controls (Fig. 4D). Given the drastic reduction in GR expression at both the mRNA and protein levels and the absence of defective allele expression, we suspected that the mutated GR protein was not expressed *in vivo*. Indeed, the 50-kDa mutated GR species was undetectable by western blot whereas a 50% reduction in the 90-kDa WT GR molecule was observed (Fig. 4E). Finally, owing to this decrease in the functional GR concentration in the proband's fibroblasts, significantly below-normal induction of FKBP5, a glucocorticoid-induced target gene [16], was observed after DEX exposure (Fig. 4F).

Altogether, these findings unambiguously establish that the heterozygous nonsense mutation p.R469[R,X] results in GR haploinsufficiency that ultimately compromises glucocorticoid signaling *in vivo*.

## Discussion

We describe a family carrying the first heterozygous nonsense mutation (R469X) in the GR gene. This germinal mutation results in partial glucocorticoid resistance associated with subclinical hypercortisolism, bilateral adrenal hyperplasia and hypertension but yet low mineralocorticoid levels, aldosterone and deoxycorticosterone, defining an apparent mineralocorticoid excess. *In vitro* characterization revealed that the GR p.R469X mutant is unable to bind hormones and DNA. GR p.R469X mutant does not exhibit any ligand-dependant nuclear translocation nor display transcriptional activity. *In vivo* functional characterization of the endogenous heterozygous nonsense mutation demonstrated that the molecular mechanism underlying glucocorticoid insensitivity in this kindred involves GR haploinsufficiency and nonsense-mediated mRNA Decay (NMD) [17]. This mechanism of glucocorticoid signaling dysfunction differs from that induced by other heterozygous missense GR mutations that impair one or several steps of the GR activation cascade and/or exert dominant negative effect on wild-type GR [6].

The rare familial or sporadic cases of primary glucocorticoid resistance [6] are associated with mild to very severe clinical presentations. In this family, the clinical presentation was quite unusual. Indeed, adrenal incidentalomas are emerging as an increasingly important clinical entity owing to the routine use of efficient imaging techniques. Its prevalence increases with age, affecting as much as 10% of the elderly [11]. Among clinically unapparent adrenal masses, bilateral adrenal hyperplasia often causes difficulties of diagnostic and management. Investigation of adrenal incidentalomas leads to the identification of this nonsense mutation in eight individuals spanning three generations allowing us to examine the natural history of the disease and to study the effects of human GR deficiency throughout the life cycle. Moderate hypercortisolism is associated with an almost normal phenotype with normal fertility and no virilization in affected women. A striking observation is the extent of bilateral adrenal hyperplasia which progresses gradually, probably owing to chronic exposure to inappropriate ACTH levels; this clinical presentation resembles that of heterozygous GR^+/−^ mice [10]. Alternatively, altered intraadrenal glucocorticoid-regulated adrenocortical cell signaling [18] could play a prominent role in the pathogenesis of the disease.

Besides GR, glucocorticoids also bind the closely related mineralocorticoid receptor (MR) [5]. Therefore, despite a drastic reduction in normal GR abundance, longstanding glucocorticoid excess may have deleterious effects through MR binding. In classical aldosterone target tissues such as the distal nephron, the enzyme 11βHSD2 metabolizes cortisol into inactive cortisone [13] and prevents permanent MR activation with subsequent sodium retention and arterial hypertension. Indeed, the THE/THF ratio was low and fell gradually over the generations in this kindred, strongly suggesting impaired activity of renal 11βHSD2, a direct GR target gene [19]. Owing to the absence of elevated aldosterone and DOC levels, increased cortisol levels induce illicit occupation and activation of the unprotected MR leading to an apparent mineralocorticoid excess with hypertension and hypokalemia. Glucocorticoid-activated MR could also trigger proadipogenic effects [20], or affect neuron function [21], although this needs to be investigated in patients with glucocorticoid receptor haploinsufficiency.

On the other hand, the absence of hyperglycemia or hyperlipidemia in the propositus despite chronically high glucocorticoid levels, suggests that the reduction in hepatic or adipose GR levels might somehow protect against glucose intolerance or metabolic alterations as observed in equivalent animal models [10], [22], [23]. It remains to be established how and to which extent such an unbalanced GR dosage affects metabolic, central nervous system or cardiovascular functions in these affected individuals.

In conclusion, our results provide first evidence for a new human genetic defect due to nonsense-mediated mRNA Decay responsible for GR hapoinsufficiency. Indeed, GR haploinsufficiency identified in this family compromises glucocorticoid sensitivity and may represent a novel genetic cause of subclinical hypercortisolism, incidentally revealed bilateral adrenal hyperplasia and mineralocorticoid-independent hypertension. We propose that a GR genetic screening should be proposed and particularly relevant in such patients given the possible therapeutic potential of MR antagonists [24], [25].

## Materials and Methods

### DNA analysis

All the participants gave their written informed consent for genetic analyses which were approved by the local ethics committee (CHU Bicêtre). Genomic DNA was extracted from white blood cells. The entire coding regions of the hGRα and hGRβ genes were amplified and sequenced with primers described in Supplemental Table S1.

### Mutagenesis

Arginine to stop mutation (hGRα-R469X) was obtained with the QuickChange Site-Directed Mutagenesis Kit (Stratagene, La Jolla, CA) with pcDNA3-hGRa as a matrix.

### Cell culture

Fibroblasts (passages 5–15) were cultured at 37°C with 5% CO_2_ in DMEM High Glucose (4.5 g/l), 2 mM glutamine, 1 mM sodium pyruvate, 100 U/ml penicillin, 100 mg/ml streptomycin and 10% fetal calf serum, pH 7.4.

### RT-PCR and quantitative real-time PCR

Total RNA was extracted from cells with Trizol reagent (InVitrogen, Cergy Pontoise, France) and gene expression was quantified by real-time RT-PCR, using an ABI 7300 Sequence Detector (Applied Biosystems, Foster City, CA) as described [26].

### Transcription assays

HEK 293 cells starved for 24 h in steroid-free medium were transfected using Lipofectamin 2000 (InVitrogen) with pcDNA3-hGRα or pcDNA3 hGRα-R469X together with pSVbgal and pGRE2-TATA-luc plasmids and then exposed to 100 nM DEX. β-galactosidase and luciferase activities were assayed as described elsewhere [27].

### In vitro translated GR proteins

*In vitro* translated WT and mutated GR were prepared by using the TnT-T7 Quick Coupled Transcription/Translation kit (Promega, Charbonnières-les-Bains, France), and labeled with [^35^S]-methionine (Perkin Elmer, Courtaboeuf, France).

### Western blot analysis

Total protein extracts were prepared from cells lysed at 4°C, as previously described [26]. Immunoblots were incubated overnight with anti-GR antibody (AbC10-G015, AbCys Paris, France) followed by a peroxidase-conjugated goat anti-mouse antibody (Vector, Burlingame, CA) for 30 min at room temperature. Proteins were visualized with the ECL^+^ detection kit (GE Heathcare, Buckinghanshire, UK).

### Binding assays

Primary cultures of patient fibroblasts were grown for 24 h in minimal medium (DMEM High Glucose), then incubated for 1 h at 37°C with 50 nM [^3^H]-DEX (1517 GBq/mmol; Perkin Elmer) in the presence or absence of 25 mM unlabeled DEX. Specific [^3^H]-DEX binding was determined as the difference between total and nonspecific binding and was expressed as sites/cell.

### Gel retardation assay

Electromobility shift assays were essentially performed as previously described [26]. Purified complementary oligonucleotides (GRE-forward: 5′-AGCTGCTCAGCTAGAACACTCTGTTCTCTACT-3′ and GRE-reverse 5′-AGCTAGTAGAGAACAGAGTGTTCTAGCTAGC-3′) were annealed and radiolabeled with [^32^P]-dCTP (Perkin-Elmer) using the Klenow fragment of DNA polymerase (Invitrogen) to a specific activity of approximately 10^8^ cpm/µg of DNA. *In vitro* translated wildtype and mutated GR were incubated with radiolabeled GRE probe for 15 min at 25°C in the absence or presence of 100-molar excess of labeled GRE and separated from free dsDNA by non-denaturing electrophoresis in a 4.5% acrylamide/bisacrylamide (37∶1) gel for 1 h at 200V (40 mA) in 0.25× TBE (50 mM Tris, 50 mM boric acid, 1 mM EDTA). Gels were dried and exposed to X-ray film at −80°C.

### Immunocytochemistry

WT and mutated GR transfected cells were grown on acid-etched and poly-d-lysine-coated glass coverslips. After DXM treatment, cells were rinsed in ice-cold PBS, fixed with a 4% formaldehyde solution in PEM buffer (0.1 M PIPES, 2 mM EGTA, 3 mM MgCl2), permeabilized in 0.5% Triton X-100 in PEM buffer for 30 min, and quenched in sodium borohydride (0.5 mg/ml in PEM buffer). Coverslips were then incubated for 1 h (RT) in 5% nonfat dry milk in TBST before overnight incubation at 4°C with anti-GR antibody (AbC10-G015) and subsequently with goat anti-mouse antibody-Alexa 555 (Molecular Probes). Cells were postfixed and counterstained with DAPI (4′,6′-diamidino-2-phenylindole) (0.5 mg/ml for 1 min), rinsed in water, and mounted onto slides (ProLong Gold; Molecular Probes).

### Statistical analysis

We used nonparametric Mann-Whitney test and Kruskal-Wallis multi-variance analysis followed by a post-test analysis of Dunn's comparison test (Prism 4, GraphPad Software, San Diego, CA).

## Supporting Information

Figure S1 Genotyping of the heterozygous nonsense mutation R469[R,X] of the glucocorticoid receptor. A) Sequencing of exon 4 in genomic DNA (gDNA) of individual II.1 lymphocytes confirmed the existence of the normal GR coding region. B and C) Identification of the heterozygous 1405C>T transition. Sequencing of exon 4 in genomic DNA (gDNA) of lymplocytes (B) and fibroblasts (C) of patient II.3 DNA indicated that the proband was heterozygous for a single C>T nucleotide change at position 1405, converting the amino acid arginine (R) into a premature stop codon (X) at position 469 of GR in all affected individuals.
(4.02 MB TIF)

Table S1 Primer sequences of hGR (NR3C1) for PCR, and sequencing. The sense and antisense primers were designed, GC% calculated and Tm estimated with the online version 0.4 of Primer 3. All primers were blasted to check their selectivity on the NCBI web site using the "Human Genomic Plus Transcript database". The sequences in bold indicate exonic primers while the others are intronic. cDNA2-5 refers to the amplified cDNA fragment encompassing exon 4.
(0.07 MB DOC)
