# Supplementary figures and images for: Familial Glucocorticoid Receptor Haploinsufficiency by Non-Sense Mediated mRNA Decay, Adrenal Hyperplasia and Apparent Mineralocorticoid Excess
Source: PLoS One. 2010 Oct 22;5(10):e13563. doi: 10.1371/journal.pone.0013563 (PMC2962642; doi:10.1371/journal.pone.0013563)

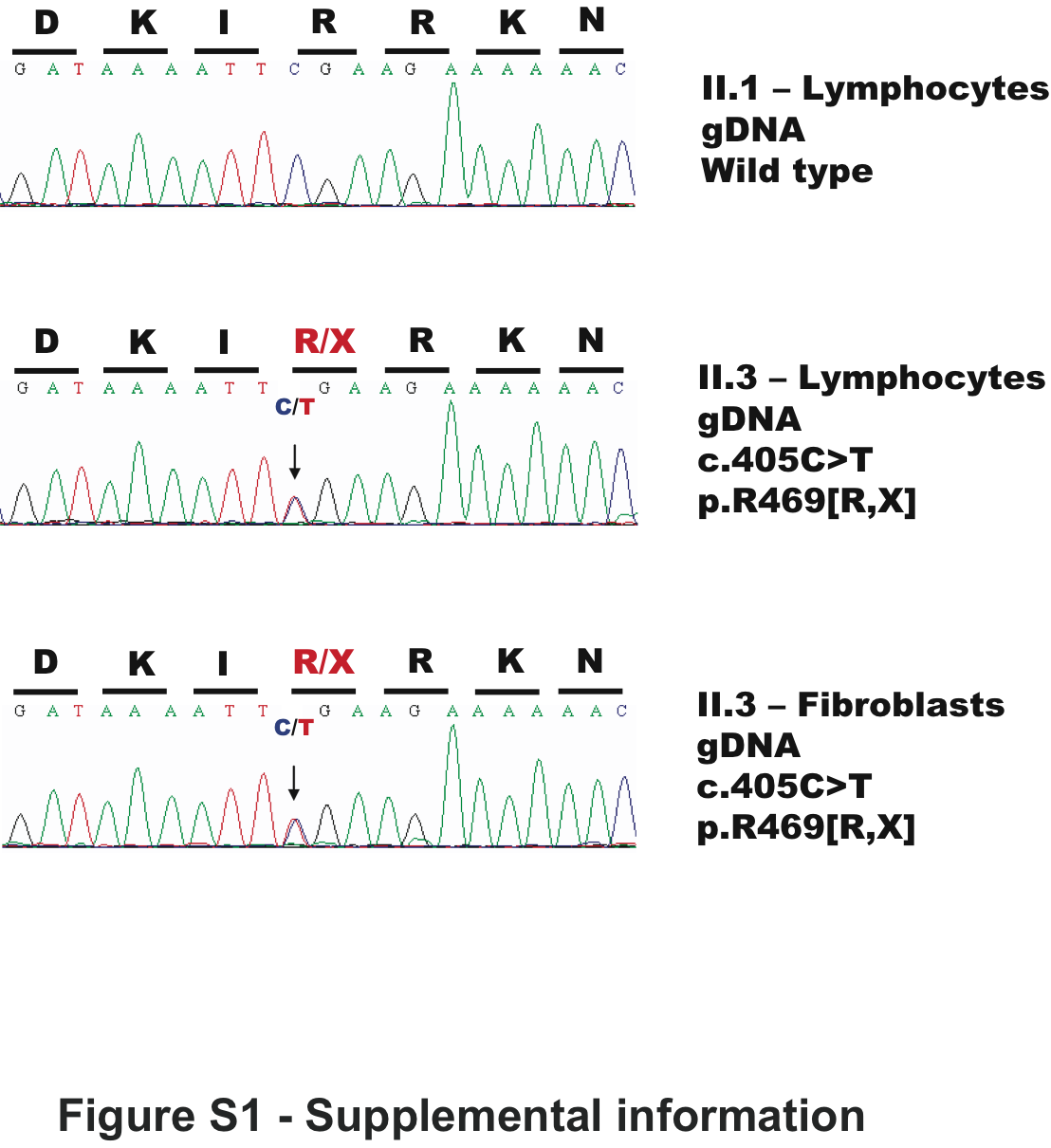

Supplement: Figure S1 — Genotyping of the heterozygous nonsense mutation R469[R,X] of the glucocorticoid receptor. A) Sequencing of exon 4 in genomic DNA (gDNA) of individual II.1 lymphocytes confirmed the existence of the normal GR coding region. B and C) Identification of the heterozygous 1405C>T transition. Sequencing of exon 4 in genomic DNA (gDNA) of lymplocytes (B) and fibroblasts (C) of patient II.3 DNA indicated that the proband was heterozygous for a single C>T nucleotide change at position 1405, converting the amino acid arginine (R) into a premature stop codon (X) at position 469 of GR in all affected individuals. (4.02 MB TIF) [file pone.0013563.s001.tif]
