# Supplementary material for: Familial Glucocorticoid Receptor Haploinsufficiency by Non-Sense Mediated mRNA Decay, Adrenal Hyperplasia and Apparent Mineralocorticoid Excess
Source: PLoS One. 2010 Oct 22;5(10):e13563. doi: 10.1371/journal.pone.0013563 (PMC2962642; doi:10.1371/journal.pone.0013563)
Supplement: Table S1 — Primer sequences of hGR (NR3C1) for PCR, and sequencing. The sense and antisense primers were designed, GC% calculated and Tm estimated with the online version 0.4 of Primer 3. All primers were blasted to check their selectivity on the NCBI web site using the "Human Genomic Plus Transcript database". The sequences in bold indicate exonic primers while the others are intronic. cDNA2-5 refers to the amplified cDNA fragment encompassing exon 4. (0.07 MB DOC) [file pone.0013563.s002.doc]

| **Name** | **Ampl. size** | **Sense primer** | **Antisense primer** | **Length** | **Tm** | **%GC** |
| --- | --- | --- | --- | --- | --- | --- |
| Exon1-1 | 500 bp | ttttctggcacctgcttgat | **TCATGCTCAACAAGGATTTGA** | 20/21 | 61/59°C | 45/38% |
| Exon1-2 | 496 bp | **GAAGGTCAGAAATCTTTCAAGC** | **TGACTTAGATATGACGCAGATTCC** | 22/24 | 57/60°C | 41/42% |
| Exon1-3 | 590 bp | **CAGTGGCTCTGAGCTGAAC** | tgatgctctgacctctgagtg | 20/21 | 59/61°C | 55/57% |
| Exon2-1 | 595 bp | caagctgcctcttactaatcg | **AAGATACATCAGAGTGAGTTTTTGGA** | 21/26 | 57/60°C | 48/35% |
| Exon2-2 | 599 bp | **GAAAGCATTGCAAACCTCAA** | **CCATGAACAGAAATGGCAGA** | 20/20 | 59/60°C | 40/45% |
| Exon2-3 | 593 bp | **GCCTCTCATTTTACCGGACA** | ttcctactttcaaaaggccact | 20/22 | 60/59°C | 50/41% |
| Exon3 | 390 bp | tgctagcacttgaagccaga | ttagcctttcatgggctttg | 20/20 | 60/60°C | 50/45% |
| Exon4 | 287 bp | accggaaacaaagacagagg | catctgcttacgtgtatcttcaaaa | 20/25 | 59/60°C | 50/36% |
| Exon5 | 496 bp | ttgctaaataaaatatttctcccatc | ccatgggctcacgatgata | 26/19 | 58/60°C | 27/53% |
| Exon6 | 388 bp | ttgatctcattgctccttgg ttgatctcattgctccttgg | gccccaagcactcataactc | 20/20 | 59/60°C | 45/55% |
| Exon7 496 bp | 496 bp | taaacagccaagatgcagga | tggtgtcacttactgtgccttt | 20/22 | 59/60°C | 45/45% |
| Exon8 | 483 bp | aaggaggtcaaggctccagt | ttggccttaggaaaaatgtct | 20/21 | 60/58°C | 55/38% |
| Exon9α 591 bp | 591 bp | tttttcaaaatagaggacaacaaa | **AAAAGTATGAAGAGAAAGTTCATCACA** | 24/27 | 57/59°C | 25/30% |
| Exon9β | 448 bp | **TGTAACCCGGCTGGATAAAT** | **CAACAGTTTGGGTTGGGATG** | 20/20 | 59/61°C | 45/50% |
| cDNA2-5 | 589 bp | **GCCAAGGATCTGGAGATGAC** | **AGCGTAGTCATGATCCTCCAA** | 20/21 | 60/60°C | 55/48% |

#

### **Supplemental Table 1 SI**: **Primer sequences of hGR (NR3C1) for PCR, and sequencing**

The sense and antisense primers were designed, GC% calculated and Tm estimated with the online version 0.4 of Primer 3. All primers were blasted to check their selectivity on the NCBI web site using the “Human Genomic Plus Transcript database”. The sequences in bold indicate exonic primers while the others are intronic. cDNA2-5 refers to the amplified cDNA fragment encompassing exon 4.
